# Distribution of immunodeficiency fact files with XML – from Web to WAP

**DOI:** 10.1186/1472-6947-5-21

**Published:** 2005-06-26

**Authors:** Jouni Väliaho, Pentti Riikonen, Mauno Vihinen

**Affiliations:** 1 Institute of Medical Technology, FI-33014 University of Tampere, Finland; 2 Department of Information Technology, University of Turku, FI-20520 Turku, Finland; 3 Research Unit, Tampere University Hospital, FI-33520 Tampere, Finland

## Abstract

**Background:**

Although biomedical information is growing rapidly, it is difficult to find and retrieve validated data especially for rare hereditary diseases. There is an increased need for services capable of integrating and validating information as well as proving it in a logically organized structure. A XML-based language enables creation of open source databases for storage, maintenance and delivery for different platforms.

**Methods:**

Here we present a new data model called fact file and an XML-based specification Inherited Disease Markup Language (IDML), that were developed to facilitate disease information integration, storage and exchange. The data model was applied to primary immunodeficiencies, but it can be used for any hereditary disease. Fact files integrate biomedical, genetic and clinical information related to hereditary diseases.

**Results:**

IDML and fact files were used to build a comprehensive Web and WAP accessible knowledge base ImmunoDeficiency Resource (IDR) available at . A fact file is a user oriented user interface, which serves as a starting point to explore information on hereditary diseases.

**Conclusion:**

The IDML enables the seamless integration and presentation of genetic and disease information resources in the Internet. IDML can be used to build information services for all kinds of inherited diseases. The open source specification and related programs are available at .

## Background

Biomedical information is often very complex. Deciphering the roles of genes in human health and disease is a grand challenge for many reasons, including impediments to defining phenotypes, difficulties in identifying and quantifying environmental effects, technical problems in generating genotypic information, and the difficulties of studying humans [1]. The completion of the draft sequence of the human genome [2,3] and advances in molecular biology provide new opportunities to increase our understanding of the role of genetic factors in human health and disease [1]. The number of identified genetic diseases has increased exponentially [4]. The new knowledge can be applied to the prevention, diagnosis and treatment of diseases. This far, the knowledge of genetics has had a large role in the health care of only a few patients and a small role in the health care of many [5]. The biomedical informatics holds great promise for developing informatics methods that will be crucial in the development of genomic medicine [6].

Most hereditary diseases are rare and the diagnosed patients for a condition are often randomly spread out in the world. One doctor usually has only a few patients with a disease. It is often difficult to find comprehensive and validated biomedical information related to rare diseases. In addition, it is more and more difficult to publish results in scientific journals only from a few cases even when they are interesting [7]. Still, all these pieces of information can contain clues to understanding the fundamental defects at molecular level and can help to develop targeted treatments. The scattering of the disease-related information to literature and Internet is a big obstacle especially for those interested in rare diseases. First of all, there may not be that much data for these diseases and secondly it may be very difficult to find and collect. Further, the user has often difficulties in assessing the quality of data.

There is an increasing need for tools and services capable of integrating information from a variety of sources. Clinicians and researchers could benefit from a more consolidated and unified view of the available biomedical data. Systems biology researchers need to integrate disparate information from multiple public sources to merge with their own experimental data to generate models of processes. Biomedical data mining attempts to extract information from biomedical databases by using e.g. automated natural language processing (NLP) techniques [8]. Processing of biomedical texts presents many challenges such as in the areas of terminology or ontology building, information extraction from texts, knowledge discovery from collections of documents, as well as sharing and integrating knowledge from factual and textual data bases, semantic annotation, etc. Without standardized nomenclature the information extraction (IE) about a particular subject from various resources is difficult. Due to ambiguity of terms, a search for a particular term often retrieves results for unrelated entities. Since there are also some technical problems arising from the diversity of computer hardware and software, there is a need for such a data form, that can be handled by any computer and which can be easily presented on any platform.

The Extensible Markup Language (XML) is a standard created by the World Wide Web Consortium (W3C) for characterizing the content and structure of documents [9]. It is designed to improve the functionality of the Web by enabling more flexible and adaptable information identification and presentation. XML allows to define tags and document structures for own context-specific use. It was derived from SGML (Standard Generalized Markup Language), the international standard for defining descriptions of the structure and content of different types of electronic documents [10]. XML is simpler than SGML, but it allows the use of richly structured documents over the Internet. Information encoded in XML is easy to read and understand, and easy to process by computers. In XML files, structured data are bounded by tags and attributes. XML tags, attributes and element structure provide context information that facilitates the interpretation of the meaning of content, thereby making it feasible to develop efficient search engines and agents and perform intelligent data mining, etc. The XML allows the separation of content, logic and presentation.

Beyond XML there are a number of additional specifications such as Document Object Model (DOM) [11], XML Schemas [12], XSL Transformations [13], and Resource Description Framework (RDF) [14]. XML will have a big role in integration and interoperation of biological databases. Some biomedical information models have been implemented using XML specifications [15,16], many of them being clinical models for electronic healthcare documents [17-19].

A unified data format of resources is required for comparison between similar diseases and reutilization of information. Here we present a new data model called fact file, which integrates biomedical information related to hereditary diseases into a Web and WAP accessible knowledge base. Our scope is wider than e.g. in gene oriented knowledge bases such as GeneCards [20], UniGene [21], or LocusLink [22]. The disease information sources are even more diverse than those for genetic information. The fact files concentrate on sharing and integrating biomedical knowledge from different sources. The presented data model can be applied to any hereditary disease.

The fact files were applied to build a comprehensive, validated knowledge base for primary immunodeficiencies (PIDs) called ImmunoDeficiency Resource (IDR) [23,24]. It is designed for different user groups such as researchers, physicians and nurses as well as patients and their families and the general public. The IDR is the major information source to immunodeficiencies in the Web. Fact files serve as the core of the IDR knowledge base.

## Methods

**Figure 1:**
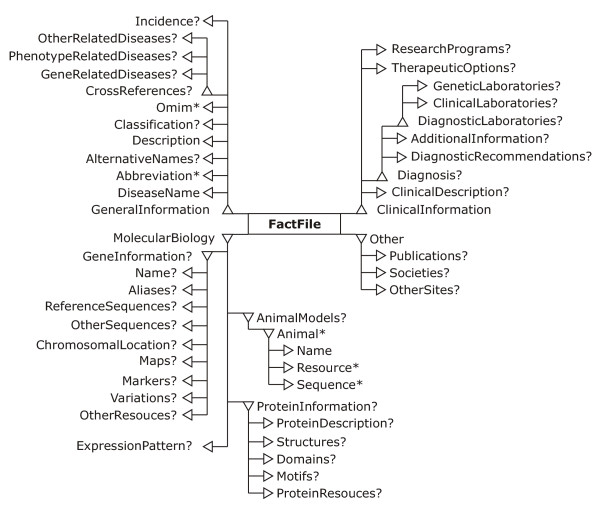
**The tree diagram of main IDML elements of fact file data model. **The operators "?" (optional), "*" (zero or more) and "+" (one or more) are used to denote cardinality indicating how many instances of an element type are permitted.

The fact file data model and the Inherited Disease Markup Language (IDML) were developed to facilitate disease information integration, storage and exchange in the first place for immunodeficiencies, but in principle for any hereditary disease. The IDML is an XML specification and container for bioinformatical data on hereditary diseases. The fact file data model schema was defined according to W3C XML specification [12, see Additional files 1, 2, 3]. The fact file data model can be depicted as a tree structure graph where a <*FactFile*> element is a root (Figure 1). Fact files make use of the following specifications, standards and databases: HUGO nomenclature [25], RefSeq [22], Swiss-Prot [26] and SOURCE [27].

Stand-alone IDML fact files have been generated for each PID. The fact files are uniquely identified by an *id *attribute of *FactFile *root element. The major concepts in the first tier of the fact file hierarchy below the root level are general information, clinical information and molecular biology (Table 1). In addition, there are other resources, which contain links to related information providers. Each of these elements, in turn, comprises one or more additional levels of guideline constructs.

**Table 1:** The description of high-level concepts in the fact file document model

**Element**	**Description**	**Content**	**Content model^a^**
**FactFile**	The root element for IDML-based fact file document	Elements	(GeneralInformation, ClinicalInformation, MolecularBiology, Other)
**GeneralInformation**	Describes the disease in general terms	Elements	(DiseaseName, Abbreviation*, AlternativeNames?, Description, Classification?, Omim*, CrossReferences?, Incidence?)
**ClinicalInformation**	The short overview of characteristic clinical features	Elements	(ClinicalDescription?, Diagnosis?, TherapeuticOptions?, ResearchPrograms?)
**MolecularBiology**	Molecular genetic elements	Elements	(GeneInformation?, AnimalModels?, ProteinInformation?, ExpressionPattern?)
**Other**	Other related information	Elements	(Publications?, Societies?, OtherSites?)

^a ^Content model consists of a set of parenthesis enclosing some combination of child element names and operators. The order operators "," (strict sequence) and "|" (choice) indicate how elements may be combined. The operators "?" (optional), "*" (zero or more) and "+" (one or more) are used to denote cardinality indicating how many instances of an element type are permitted?

The components of the fact file model are defined as IDML elements. According to XML, elements have distinct names and they are delimited with start and end tag, e.g. <*DiseaseName*>X-linked agammaglobulinemia</*DiseaseName*>. Elements may contain other elements or attributes, they may store text, or they may be empty. Elements may appear as often as required. Many IDML elements contain *href *attribute for hyperlinking to more detailed information by using globally unique idenfier URL (Unified Resource Locator). The element naming convention follows the approach used by Electronic Business XML (ebXML) core components [28]. The IDML specified element names are in upper camel case (*UpperCamelCase*) and attribute names are in lower camel case (*lowerCamelCase*) notations. The usage of acronyms has been avoided, but when they are used the capitalization remains (example: *ReferenceDNA*).

General information elements identify a particular genetic disease and describe pattern of heritance and frequency in general terms (Table 2). The <*Abbreviation*> element includes commonly used abbreviations and the <*AlternativeNames*> element lists known aliases and synonymous names for the disease. The <*Description*> element provides a short overview in general terms. The description text may contain several *Glink *-tagged words that can act as links to a glossary, which is an integral part of the IDR service. The <C *lassification*> element is used to classify a disease explicitly to a group of related diseases. It exploits the hierarchic structure of XML documents by nesting <*Class*> elements. Each <*Class*> element contains a unique identifier in *level *attribute and a class name in <*Title*> element. The <*Omim*> element links the fact file to the Online Mendelian Inheritance in Man (OMIM) knowledge base [4] and the <*CrossReferences> *element refers to the related fact files grouped in <*Phenotype*>, <*Gene*>, and <*OtherRelatedDiseases*> elements. The incidence element stores information about disease frequency in human populations.

**Table 2:** The description of IDML: GeneralInformation element

**Element**	**Description**	**Content**	**Content model^a^**
**DiseaseName**	Disease name	Type	String
**Abbreviation**	Abbreviation for disease name	Type	String
**AlternativeNames**	List of alternatively used disease names	Elements	(Name*)
**Description**	General description of disease	Mixed	(Glink | Italic)*
**Classification**	Classifies document explicitely in the fact files hierarchy	Elements	(Class)
**Omim**	A collection of the related references to the OMIM database	Elements	(OmimReference+)
**CrossReferences**	Refers to the related fact files	Elements	(PhenotypeRelatedDiseases?, OtherRelatedDiseases?, GeneRelatedDiseases?)
**Incidence**	Description of incidence	Type	String

^a ^See table 1

Clinical information elements provide a short overview of characteristic clinical features, diagnosis, treatment and research related to the disease (Table 3). The <*ClinicalDescription*> element stores text, that describes characteristic clinical features and the most important laboratory findings. The <*Diagnosis*> refers to data on diagnostic criteria and guidelines. It also refers to databases of laboratories performing clinical and/or genetic analyses for the disease including IDdiagnostics [29], the European Directory of DNA diagnostic Laboratories (EDDNAL, ) and GeneTests [30]. Detailed diagnostic guidelines are available for several IDs [31]. The <*TherapeuticOptions*> lists therapeutic interventions that are available. The <*ResearchPrograms*> includes important research and clinical trials related to the disease.

**Table 3:** The description of IDML: ClinicalInformation element

**Element**	**Description**	**Content**	**Content model^a^**
**ClinicalDescription**	Describes characteristic clinical features	Mixed	(Glink | Italic)*
**Diagnosis**	A collection of diagnostic guidelines and laboratories	Elements	(DiagnosticRecommendations?, AdditionalInformation?, DiagnosticLaboratories?)
**TherapeuticOptions**	A collection of available therapeutic options	Elements	(Option+)
**ResearchPrograms**	A collection of related studies		(Program+)

^a ^See table 1

Molecular biology comprises the main genetic components on DNA, RNA and protein level, animal models, protein properties and expression patterns (Table 4). The <*GeneInformation*> elements store the basic information on gene names, aliases and synonyms. They provide also <*ReferenceSequences*> element that covers reference sequences on three levels and lists also other related sequences that are available from sequence databanks. Information on gene locus is stored in <*ChromosomalLocation*>, <*Maps*>, and <*Markers*> elements. The <*Variations*> element refers to related locus specific mutation and single nucleotide polymorphism (SNP) databases. We and others are maintaining a large number of immunodeficiency mutation databases [24,32]. The <*OtherResources*> element refers to the other genetic web services such as Ensembl [33], GENATLAS [34], GeneCards [20], UniGene [21], LocusLink [22], euGenes [35], GDB [36], GeneLynx [37] and SOURCE [27]. The <*AnimalModels*> element refers to the related transgenic animal studies.

**Table 4:** The description of IDML: MolecularBiology element

**Element**	**Description**	**Content**	**Content model^a^**
**GeneInformation**	Contains information on the gene name, aliases, reference sequences, chromosomal location, maps, markers, variations and other gene related resources	Elements	(Name?, Aliases?, ReferenceSequences?, OtherSequences?, ChromosomalLocation?, Maps?, Markers?, Variations?, OtherResources?)
**AnimalModels**	A collection of related transgenic animal studies	Elements	(Animal*)
**ProteinInformation**	Contains information on protein characteristic features, structures, domains, motifs and other protein resources	Elements	(ProteinDescription?, Structures?, Domains?, Motifs?, ProteinResources?)
**ExpressionPattern**	Gene expression levels in a variety of cells and tissues	Elements	(Expression*)

^a ^See table 1

The <*ProteinInformation*> element stores characteristic structural and functional properties of the protein. The <*ProteinDescription*> contains several subelements e.g. <*Function*>, <*SubcellularLocation*>, <*CatalyticActivity*>, which are inherited from the Swiss-Prot entry model [26]. The <*Structures*> element refers to solved protein structures available in Protein DataBank (PDB) [38]. The domain and motif elements describe conserved protein regions. Each <*Domain*>, <*Motif*> and further <*ProteinResources*> element includes links to related resources for example in Pfam [39], InterPro [40], ProDom [41], SMART [42] or PROSITE [43]. The <*ExpressionPattern*> stores information on gene or protein expression. This information is mainly from SOURCE [27], which is a web-based resource bringing together genetic information from different sources.

The last high level element <*Other*> stores various information in elements such as <*Publications*> and <*Societies*>, which is categorized by <*GeneralSocieties*> and <*DiseaseSpecificSocieties*> elements (Table 5). The <*OtherSites*> element refers to other related resources in the Internet.

**Table 5:** The description of IDML: Other element

**Element**	**Description**	**Content**	**Content model^a^**
**Publications**	A collection of related publications	Elements	(PubmedSearch?, Pubmed?)
**Societies**	List of related general and disease specific societies	Elements	(GeneralSocieties?, DiseaseSpecificSocieties?)
**OtherSites**	A collection of other related Web sites	Elements	(Site+)

^a ^See table 1

The IDML schema version 1.0 (idml.xsd file), examples of IDML-document and documentation on the syntax are available at our web site . The IDML document type definition file (idml.dtd) is also available, althougth we prefer to use the IDML schema for validation. Many IDML elements are optional. The syntax allows one to put comments, both within and outside of the XML markup. The parser must pass internal comments to the application programs, which can then properly treat the information. IDML documents specify which version of the schema is to be used to validate their content, eliminating possible confusion when several versions exist. IDML is open access, however, a licence is needed for building other services. Contact the authors for details.

## Results

**Figure 2:**
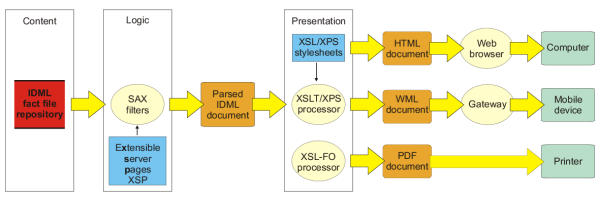
**The data flow diagram of IDR. **Notations: A red open-ended rectangle represents data store, light yellow ovals represent processes, blue rectangles represent external entities, orange rounded rectangles shows transferred documents, green rounded rectangles represent destination devices and arrows shows the flow of information from its source to its destination. White rectangles show the separation of content, logic and presentation.

The IDML model was implemented to describe primary immunodeficiencies, which is a group of over 100 hereditary diseases. IDs can be grouped as follows: combined B and T cell immunodeficiencies, deficiencies predominantly affecting antibody production, defects in lymphocyte apoptosis, other well-defined immunodeficiency syndromes, defects of phagocyte function, interferon-γ (IFNγ) associated immunodeficiencies, DNA breakage associated syndromes, defects of the complement cascade proteins, and defects of complement regulatory proteins. The disease information is stored in IDML-based fact files, which form the central repository for data retrieval of ImmunoDeficiency Resource (IDR) service [23,24,29] available at . The data flow diagram of IDR is shown in figure 2. In addition to information on fact files, the IDR contains several introductory texts and collections of immunology related data sources. The IDR pages are extensively hyperlinked to our on-line immunology glossary. More detailed description about the IDR web service has been published elsewhere [23,24].

The ImmunoDeficiency Resource is a comprehensive knowledge base on immunodeficiencies. IDR is developed and maintained by IMT Bioinformatics group in collaboration with experts on individual immunodeficiencies. All the information in the IDR will be validated by expert curators. However, all changes, additions and corrections to the fact files are made by our group. IDR is designed for different user groups such as researchers, physicians and nurses as well as patients and their families and the general public. IDR contains fact files for practically all known PIDs. The numerous individual data items in IDR have been collected partly manually, usually with simple Perl scripts written for datamining from numerous local and Internet databases and services.

We selected Apache AxKit XML Application Server version 1.61 for implementation of the IDML-encoded web service. AxKit is an application and document server that uses XML processing pipelines to generate and process content and to deliver it to clients in a wide variety of formats, such as HTML, WML, PDF and plain text using either standard techniques of World Wide Web Consortium (XSLT) [13], or flexible custom codes (XPathScript XPS, eXtensible Server Pages XSP).

Similar XML application server called Cocoon, has been written in Java. We settled on AxKit, because it is built in Perl, which makes it easy to integrate with bioinformatic applications many of which are written in Perl. It is important to note that AxKit is not limited to XML source documents. Non-XML documents and data sources can be converted to XML when necessary. AxKit separates the content, logic and presentation. The content reuse was implemented with XInclude [44] and XPointer [45] techniques. The root element of IDML schema is <*FactFiles*> and according to W3C Recommendation "Namespaces in XML" [46] we declared a default namespace attribute in the root element xmlns:idml="" to avoid the problems of ambiquity and name collisions.

Each fact file is stored in an IDML file, that has a unique name and url address. When a fact file requests the pipeline it might look like this in diagramatic terms : Request > [XSP] > (XML) > [XSLT] > (HTML) > Browser, where processors are in square brackets and products in round brackets. The output of XSP pages is structured XML content, which can pipe through XSLT to produce HTML. The XSP feature is not currently in use in the IDR.

The information on fact files can be easily transformed and presented in any platform. It is easy to write platform or even browser and screen specific pages. We have implemented a transformation from IDML to WML for portable devices (such as mobile phones) with WAP compliance (Wireless Application Protocol). The fact files are available via bioinformatics related WAP service, BioWAP [47,48] practically anywhere, anytime.

New web techniques are developed continuously. During this project a number of new specifications and software appeared, requiring upgrading of the system many times. The separation of content and presentation enables to share the project for people who are responsible for information content and people who develop the knowledge management techniques. Once the data model was created, we have not had to touch it hardly at all in spite of technical improvements, content additions and deletions.

## Discussion

As far as we know there are no other efforts to develop a markup language to describe connections between disease and genetic information. The IDML was designed with following purposes in mind. First, we wanted a markup that is able to present disease, clinical, diagnostic and genetic information and relations between them. Secondly, the data model structure had to be intuitive, hierarchical, flexible, but still machine and human readable. Sometimes the relatively large XML files can appear verbose for human readers, but hierarchically and logically organized structure in addition to semantic markup facilitate the interpretation of documents. Thirdly, an application and platform independent data format was needed. Its portability, extensibility and robustness are primary advantages for interoperating heterogeneous systems. The availability of open source and free tools for processing files in all major programming languages is important. The openness of source code as well as data formats and data itself allows better integration and interoperation between data resources. The IDML enables the seamless integration of genetic and disease information resources in the Internet. The data model is appropriate for the implementation of automated decision support systems such as diagnostic consultations. Fourthly, the data have to be unambiguous and validated.

A fact file is a user oriented user interface, which serves as a good starting point to explore information on hereditary diseases. For some time now, there has been many advanced search facilities in the Internet such as Google, that are able to find very fast web pages that contain given keywords. However, the web searches typically turn up innumerable completetely irrelevant "hits", requiring much manual filtering by the user. Navarro *et al*. lists some issues related to database searching and accessibility that can cause difficulties [49] including inaccurate and redundant search results, nomenclature issues, lack of internal access, non-availability of the source code, lack of customization and differing data formats. New methods are needed for improving search results.

There is an increasing number of biomedical data sources in the Internet. The Human Genome Initiative [2] and other genome research projects have generated enormous quantities of data. The genetic data is well organized in web accessible databases for example EMBL [50], GenBank [51], Swiss-Prot [26], etc. Several organizations offer public interfaces for obtaining biomedical information across a range of domains. They provide numerous tools and applications for genetic data retrieval and analysis for example with Sequence Retrieval System SRS [52] and BioPerl [53]. In addition to the sequence information, databases contain a lot of valuable information in annotations. There are also some genetic knowledge bases such as GeneCards and GeneLynx that comprise the essential information on genes. Swiss-Prot contains also some disease related annotations. The most comprehensive database on hereditary diseases is OMIM [4], which contains descriptions for known hereditary diseases.

Almost all pages in the Internet have been written in HyperText Markup Language (HTML) where it is used for style description. It provides some possibilities for simple description about a document. It is able to use special metatags that contain simple keywords or more advanced descriptions like Dublin Core Languages, but they are very little utilised and only the most sophisticated search engines can exploit them.

There are some efforts to integrate heterogeneous biomedical databases [15,54,55]. Some level of standardization is required for more automatic integration. Development of integration techniques is moving databases towards the Internet and XML-based systems [56]. In the future, Web services will use standard Internet protocols including SOAP, WSDL, and UDDI for interoperability with other resources. Thereby the flexible and expandable integration of diverse scientific tools will be achieved.

## Conclusion

The XML-based language IDML and fact file data model were developed for integrating, storing and exchanging information on inherited diseases. The IDML language and fact file model are implemented in the IDR knowledge base. The fact files can be easily transformed from IDML to any format such as HTML or WML using either standard W3C techniques or flexible custom code. The content management as well as the exchange of presentation are facilitated by separating document content and presentation. The IDML-based information system was proved to be a viable and applicable specification for inherited diseases. Numerous downloads (altogether more than 250,000) from the IDR knowledge base during the last two years have proved the applicability and adaptability of the fact file model.

## List of abbreviations

AxKit An XML Delivery Toolkit for Apache

BioWAP Bioinformatics service for portable devices

DOM Document Object Model

DTD Document Type Definition

ebXML Electronic Business XML

EDDNAL European Directory of DNA Diagnostic Laboratories

EMBL Genetic sequence database by European Molecular Biology Laboratory

GDB Genome Database

GenBank Genetic sequence database by National Center for Biotechnology Information

HTML Hypertext Markup Language

IDML Inherited Disease Markup Language

IDR ImmunoDeficiency Resource

IE Information extraction

IFNγ Interferon gamma

NLP Natural language processing

OMIM Online Mendelian Inheritance in Man

PID Primary Immunodeficiency

PDB Protein DataBank

PDF Portable Document Format

Pfam Protein Families Database

ProDom Protein Domain Database

PROSITE Database of Protein Families and Domains

RDF Resource Description Framework

SGML Standard Generalized Markup Language

SMART Simple Modular Architecture Research Tool

SNP Single nucleotide polymorphism

SOAP Simple Object Access Protocol

SOURCE Genomic resource in the Internet

Swiss-Prot Protein knowledgebase

UDDI Universal Description, Discovery, and Integration

URL Unified Resource Locator

W3C World Wide Web Consortium

WAP Wireless Application Protocol

WML Wireless Markup Language

WSDL Web Services Definition/Description Language

XML Extensible Markup Language

XPS XPathScript

XSL Extensible Style Language

XSLT XSL Transformations

XSP eXtensible Server Pages

## Competing interests

The author(s) declare that they have no competing interests.

## Pre-publication history

The pre-publication history for this paper can be accessed here:



## Supplementary Material

Additional File 1 An XML schema for IDML Click here for file

Additional File 2 A DTD file for IDML Click here for file

Additional File 3 An example file using IDML. A fact file for X-linked agammaglobulinemia. Click here for file
